# Is quality of life affected by temporomandibular disorders?

**DOI:** 10.31744/einstein_journal/2018AO4339

**Published:** 2018-11-29

**Authors:** Débora de Melo Trize, Marcela Pagani Calabria, Solange de Oliveira Braga Franzolin, Carolina Ortigosa Cunha, Sara Nader Marta

**Affiliations:** 1 Universidade do Sagrado Coração, Bauru, SP, Brazil.

**Keywords:** Temporomandibular joint disorders, Quality of life, Facial pain, Diagnosis, Temporomandibular joint, Transtornos da articulação temporomandibular, Qualidade de vida, Dor facial, Diagnóstico, Articulação temporomandibular

## Abstract

**Objective:**

To determine the impact of temporomandibular disorders in quality of life.

**Methods:**

A total of 102 volunteer patients (68 female) aged 19 to 86 years, who sought medical care in health clinics of the university and were evaluated in the period from September to December 2013. The subjects were examined according to the Research Diagnostic Criteria for Temporomandibular Disorders, using a mechanical algometer (Palpeter^®^) with standardized pressure of 0.5 and 1.0kg, and Medical Outcomes Study 36-Item Short Form Health Survey (SF-36) questionnaire, to assess quality of life. The data were tabulated for statistical analysis and the variables were correlated with the clinical findings of the temporomandibular disorders and quality of life.

**Results:**

Fifty percent of patients were positive for temporomandibular disorders and 39.2% classified as myofascial pain group. The temporomandibular disorder group was significantly associated with uncomfortable bite (p=0.0000), temporomandibular joint clicking (p=0.0001) and tooth clenching (p=0.0001). The Mann Whitney test used to analyze the SF-36 revealed that the domains of pain (mean score of 47.80%; p<0.0001) and mental health (62.67%; p<0.05) were strongly associated with temporomandibular disorders.

**Conclusion:**

The quality of life of individuals with temporomandibular disorders was negatively affected by the presence of pain and mental health disorders.

## RESUMO

**Objetivo:**

Determinar o impacto das disfunções temporomandibulares na qualidade de vida.

**Métodos:**

Foram incluídos 102 pacientes voluntários (68 mulheres) com idades entre 19 e 86 anos, que buscaram atendimento médico nas clínicas de saúde da universidade e foram avaliados no período de setembro a dezembro de 2013. Os pacientes foram examinados segundo os Critérios Diagnósticos para Pesquisa em Disfunções Temporomandibulares, usando um algômetro mecânico (Palpeter^®^) com pressão padronizada de 0,5 e 1,0kg e o questionário *Medical Outcomes Study 36-Item Short Form* Health *Survey* (SF-36), para avaliação da qualidade de vida. Os dados foram tabulados para análise estatística, e as variáveis foram correlacionadas com os achados clínicos das disfunções temporomandibulares e da qualidade de vida.

**Resultados:**

Metade dos pacientes foi positiva para disfunções temporomandibulares, sendo 39,2% deles classificados como grupo de dor miofascial. O grupo de disfunções temporomandibulares foi significativamente associado ao desconforto ao morder (p=0,0000), à crepitação da articulação temporomandibular (p=0,0001) e ao apertar dos dentes (p=0,0001). O teste de Mann-Whitney usado para analisar o SF-36 revelou que os domínios da dor (pontuação média de 47,80%; p<0,0001) e saúde mental (62,67%; p<0,05) estavam fortemente associados às disfunções temporomandibulares.

**Conclusão:**

A qualidade de vida de indivíduos com disfunções temporomandibulares foi afetada negativamente pela presença da dor e de transtornos de saúde mental.

## INTRODUCTION

Temporomandibular disorder (TMD) is defined by the American Academy of Orofacial Pain (AAOP) as a collective term, comprising a number of clinical problems involving the masticatory muscles, the temporomandibular joint (TMJ), and associated structures, with common symptoms such as pain, restricted movement, muscle tenderness and intermittent joint sounds.^(^ ^1^ ^-^ ^3^ ^)^ These clinical problems, including myofascial pain, articular disc displacement, joint pain, and TMJ degeneration, have a negative influence on individual’s physical and mental health, affecting school, work, and social activities, and lead to affective and cognitive imbalance.^(^ ^4^ ^,^ ^5^ ^)^

Multifactorial etiology of TMD has been established after many years of studies, considering many factors that vary among individuals, and can contribute to the onset of signs and symptoms related to the stomatognathic system, due to changes in normal functions.^(^ ^6^ ^-^ ^11^ ^)^ For this reason, it is hard to explain the importance of each factor as predisposing, precipitating and/or perpetuating the TMD for each subject.

Pain is known to negatively impact upon the individual that is experiencing it, affecting social functioning, physical and psychological wellbeing. TMD is considered the major cause of non-dental pain in the orofacial region, and it negatively affects the quality of life (QoL).^(^ ^12^ ^-^ ^14^ ^)^ Comprehensive measurements of TMD and its impact in life are commonly captured using QoL questionnaires.

## OBJECTIVE

To determine the impact of temporomandibular disorders in quality of life.

## METHODS

A total of 102 individuals (68 female) aging from 19 to 86 years old, seeking for medical care (not necessarily related with TMD) in health clinics of an university, were evaluated in a period from September to December of 2013. Exclusion criteria were patients with psychiatric disorders, neuropathic disorders and/or continuous use of analgesics and anti-inflammatory drugs.

The subjects were initialy evaluated about the presence or not of TMD signals and symptoms. The subjects were assessed by axis I of the Research Diagnostic Criteria for Temporomandibular Disorders (RDC/TMD) for research clinical evaluation of TMD presence by one calibrated examiner. The subjects identified with TMD were referred for treatment in the TMD clinic.

A digital caliper (DC-6^®^, Western, China) was used to take measures for questions 4 and 6. For questions 8 and 9, a mechanical algometer (Palpeter^®^, Herlev, Denmark) was used. It consists of a continuous pressure stimulus in an area, with a circular flat tip (1cm of diameter) that limits the pressure to 1.0kg or 0.5kg, allowing a standardized pressure under the muscles and a calibrated examination of the sample.^(^ ^15^ ^)^

The 1.0kg pressure value was applied in points bilaterally: on the temporal muscle (anterior, medial, and posterior), masseter (origin, body and attachment) and medial third of the trapezius muscle. The 0.5kg pressure value was applied bilaterally: in the submandibular region (medial pterygoid, suprahyoid, anterior digastric region), posterior mandibular region (stylohyoid, posterior digastric region) and TMJ lateral pole region (Table 1).^(^ ^15^ ^,^ ^16^ ^)^

**Table 1:** Research diagnostic criteria/temporomandibular disorders. Summary of clinical examination form

1. Do you have pain on the right side of your face, the left side or both sides?
2. Could you point to the areas where you feel pain?
3. Opening pattern
4. Vertical range of motion
5. Sons articulares (palpação)
6. Excursions
7. Joint sounds on excursions
8. Extraoral muscle pain: temporalis (1.0kg); masseter (1.0kg); ½ muscle trapezius (1.0kg); posterior mandibular region (0.5kg); submandibular region (0.5kg)
9. Joint pain: lateral pole (outside) (0.5kg); posterior attachment (0.5kg inside ear with finger)
10. Intraoral muscle pain, palpation with fingers: lateral pterygoid area (0.5kg); tendon of temporalis (0.5kg)

Source: Translated from: Futarmal S, Kothari M, Ayesh E, Baad-Hansen L, Svensson P. New palpometer with implications for assessment of deep pain sensitivity. J Dent Res. 2011;90(7):918-22.^(^ ^15^ ^)^

Pressure was applied three times at each site, as indicated by the RDC/TMD^(^ ^16^ ^)^ recommendations. The posterior attachment, the lateral pterygoid area and the tendon of temporalis were not palpated with the Palpeter^®^. For these, the examiner was calibrated for finger pressure similar to the mechanical algometer.

Axis I RDC/TMD diagnosis comprises three groups: Group I (GI), muscle diagnosis, with myofascial pain and myofascial pain with limited opening; Group II (GII), disk displacement, with disk displacement with reduction, disk displacement without reduction and with limited opening, and disk displacement without reduction and without limited opening; Group III (GIII), articular bone degeneration, with arthralgia, osteoarthritis and osteoarthrosis.

To evaluate the QoL, a standardized and validated questionnaire Medical Outcomes Study 36-Item Short Form Health Survey (SF-36) item was applied by the researcher,^(^ ^17^ ^)^ with 11 closed questions divided into 8 domains (Table 2). Health scores range from zero (worst health) to 100 (best health).^(^ ^17^ ^)^

**Table 2:** The Medical Outcomes Study 36-Item Short Form Health Survey items

Group 1 − physical health	Funcional capacity	Vigorous activities
		Moderate activities
		Lifting
		Climbing several flights of stairs
		Climbing one flight of stairs
		Bending, kneeling or stooping
		Walking >1km
		Walking ½ km
		Walking 100 metres
		Bathing or dressing yourself
	Physical apperance	Reduce time working
		Accomplished less
		Limited in kind
		Difficult to work
	Pain	Pain intensity
		Pain interference
	General health	Your health
		Sick easier than others
		Healthy as others
		Health will get worse
		Excellent health
Group 2 − mental health	Vitality	Full of pep
		Energy
		Worn out
		Tired
	Social aspects	Interference in social activies
		Social time
	Emotional aspects	Cut down time
		Accomplished less
		Not careful
	Mental health	Nervous
		Nothing could cheer you up
		Calm and peaceful
		Down-hearted
		Happy
Health transition	Compared to one year ago, how would you rate your health in general now?	

Source: Translated from: Ciconelli R, Ferraz M, Santos W, Meinão I, Quaresma M. Translation into Portuguese and validation of the generic questionnaire for quality of life assessment SF-36 (Brazil). Rev Bras Reumatol. 1999;39(3):143-50.^(^ ^17^ ^)^

To describe the profile of the sample (n=102) according to the study variables, frequency tables containing the absolute frequencies and percentages were calculated. To compare the TMD diagnosis with the SF-36 questionnaire results, the Mann-Whitney test was used. The χ^2^ test was used to investigate an association between the groups, and classified according to the signs and symptoms of TMD. The significance level for statistical analysis was set at 5%.

The present cross-sectional study was approved by the Ethics Committee under protocol 382.371, CAAE: 19978113.5.0000.5502. The Informed Consent was voluntarily signed by the subjects of the study.

## RESULTS

The individuals examined were classified according to three different diagnostic groups as described above. From the total sample (n=102), 51 individuals had a positive diagnosis for TMD, being 34 female (66.66%) and 17 male. The following diagnoses were not pointed out: 2 subgroups of GII and the GIII. A total of 39.2% was diagnosed with myofascial pain, or pain with a muscular origin. Only 15.18% fit into GII.

The correlation result was positive for TMD groups that reported in the interview the presence of uncomfortable bite, TMJ clicking and clenching of teeth (Table 3).

**Table 3:** Groups studied and their association with the reported temporomandibular disorders signs and symptoms

Signs and symptoms	Negative Group TMD (%)	Positive Group TMD (%)	χ^2^	p value
Temporomandibular joint clicking	31	69	14.157	p=0.00017^*^
Clenching of teeth	14	49	14.754	p=0.00012^*^
Uncomfortable bite	18	71	28.989	p=0.000000072^*^
Noises or ringing in the ears	33	71	14.179	p=0.00017^*^
Night clenching of teeth	20	43	6.5571	p=0.01^*^

*χ^2^ test, p<0.05.

TMD: temporomandibular disorders.

**Figure 1:**
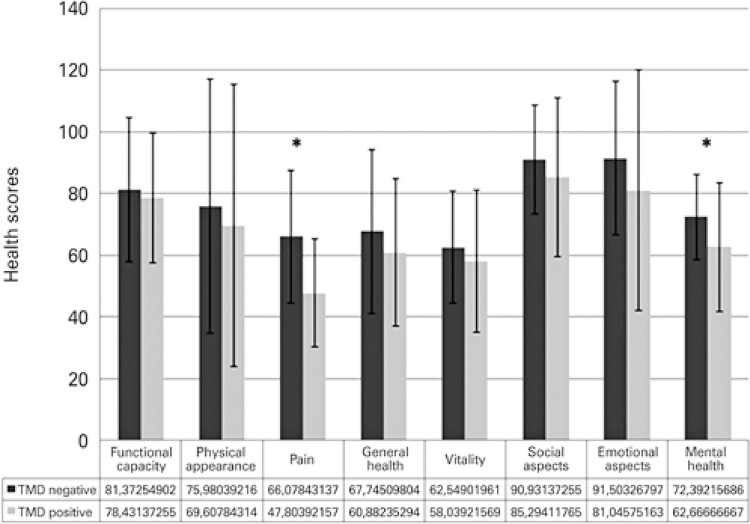
Medical Outcomes Study 36-Item Short Form Health Survey field distribution of patients with and without temporomandibular disorders

Source: produced by the author.

In the QoL evaluation, performed by the SF-36, the fields of the TMD negative group, when compared to the positive group, obtained higher scores of QoL (Figure 1). The distribution of patients in all fields showed that the TMD positive group has lower scores in all fields, despite the aspects “presence of pain” and “mental health”, that were positive and higher, compared to the group without TMD.

TMD:temporomandibular disorders.

In examining the masseter muscle bilaterally, in the TMD positive group, severe pain was expressed in 29% of subjects, which was not reported in the TMD negative group. In the TMD positive group, 35% expressed mild pain and 19% expressed moderate pain.

For the examination of the trapezius muscle, submandibular region, posterior digastric muscle frequency in severe pain was higher.

## DISCUSSION

Physical and mental health is one of the great aspects that involve a good QoL for human beings. Studying and understanding the oral and orofacial health and diseases conditions is very important for healthcare profissonals, since the disorders affecting this region can have detrimental effect on the QoL.^(^ ^18^ ^)^ The purpose of the present study was to analyze the TMD and its relation with QoL through instruments previously validated by a large amount of studies.^(^ ^4^ ^,^ ^5^ ^,^ ^19^ ^)^

Of 51 positive diagnoses for TMD, 34 (66.67%) were women. This result is consistent with several studies that also found a higher prevalence of TMD in females.^(^ ^10^ ^)^ This is attributed to an interaction of biological (differences in muscle structure and connective tissue), hormonal, psychological and social factors.^(^ ^7^ ^)^ In a prospective study,^(^ ^20^ ^)^ 6% of young adult female patients initially free of TMJ pain developed TMD. The recurrence of pain was accompanied by more frequent headache, muscle pain and other body pains. After a physical examination,^(^ ^21^ ^)^ we found that 62% of women had TMD, whereas the rate of incidence in men was only 38%. A systematic review of the literature suggested that pressure pain threshold is lower in women.^(^ ^22^ ^)^ In addition, there is strong evidence that women are less tolerant to thermal pain and to pressure pain.^(^ ^22^ ^)^

Concerning the intensity of the signs and symptoms of TMD in the present study, 39.2% were represented by the GI and 15.18%, by the GII. Similar results were found in other studies, in which TMD of myofascial pain origin presented a prevalence of 25.5%.^(^ ^8^ ^)^ Women with more than one sign or symptom related to TMD, represent 86.8% of patients seeking specialized treatment.^(^ ^23^ ^)^

In the present study, the TMD positive group presented significant association with the following reported signs and symptoms: TMJ clicking, clenching of teeth, discomfort upon biting, and noise/ringing in the ear. All these symptoms demonstrated being potencial risk factors for the development of TMD. A cohort study examined the incidence of symptoms of TMD in a period of 3 years, and showed that lesions in the TMJ, clenching of teeth and stress were significantly associated with increased risk for the development of TMD.^(^ ^6^ ^)^ Signs and symptoms of TMD, such as trauma, clenching of teeth, removal of third molar, pain somatization and female gender were associated with TMD and identified as risk factors for patients with myofascial pain.^(^ ^9^ ^)^

In the present study, uncomfortable bite, reported by 71% of patients, showed significant correlation with the TMD group. Logistic regression analysis showed that pain from TMD was associated with grinding of teeth and other parafunctional habits.^(^ ^8^ ^)^ Parafunctional habits and trauma increase frictional stress and promote a mechanical load on the TMJ. This is accompanied by the release of pro-inflammatory mediators and matrix degradation, compromising the lubrication of the TMJ, changing its properties of friction and degrading the surface of the mandibular condyle.^(^ ^24^ ^)^

The domains pain and mental health were strongly correlated with the TMD group. The presence of signs and symptoms of TMD is associated with symptoms of anxiety and depression, which would consequently affect their mental health.^(^ ^12^ ^)^ When the related QoL-related determinants of oral health were examined, the data showed their relation with psychosocial change, suggesting that these factors influence health and QoL.^(^ ^25^ ^)^

In adults, the pain of TMD has been linked to various pain points and mechanisms of peripheral and central sensitization may explain the comorbidity of these pain conditions.^(^ ^7^ ^)^ Patients with chronic TMD pain also showed greater sensitivity in other craniofacial regions and even in remote peripheral areas. This suggests that the nociceptive processing is centrally facilitated in this category of patient.^(^ ^10^ ^)^

In a randomized double blind study with women showed that in muscles analyzed bilaterally (temporal muscle, deep and superficial masseter, upper trapezius and sternocleidomastoid), there was a significant number of active and latent trigger points associated with TMD in comparison to the healthy Control Group. Thus, patients with TMD had larger areas of pain compared to those of healthy individuals.^(^ ^26^ ^)^

In the present study, in regard to the bilateral examination of the trapezius muscle, mild and severe pain were more frequent. Areas of referred pain, such as the neck, were larger than the areas of masticatory muscle pain in patients with TMD. Local and referred pain from active trigger points are similar in masticatory, neck and shoulder muscles, thus classified as spontaneous TMD, which supports the concept of peripheral and central sensitization mechanisms in myofascial TMD.^(^ ^26^ ^)^

The examination of muscle palpation and examined structures detected that the responses in relation to the right and left side were similar in proportion to laterality, which corroborates the results of a study conducted in Spain that used the same diagnostic criteria (RDC/TMD). That study showed significant differences between the groups in the areas of referred orofacial muscle pain but not between the right and left sides.^(^ ^26^ ^)^

Temporomandibular disorder was more prevalent in females, with greater incidence of myofascial pain, and similar discomfort level on both sides of the face. The QoL of TMD group subjects was affected by the presence of pain and alterations in mental health status. In the present study, TMD was associated with perception of decreased QoL.

## CONCLUSION

The quality of life of patients with temporomandibular disorders was affected by the presence of pain and alterations in the mental health. Such disorders were associated with perception of reduced quality of life.

## References

[1] Leeuw R, Kasser G (2013). Orofacial Pain: Guidelines for assessment, diagnosis, and management.

[2] Mello VV, Barbosa AC, Morais MP, Gomes SG, Vasconcelos MM, Caldas AF (2014). Temporomandibular disorders in a sample population of the Brazilian Northeast. Braz Dent J.

[3] Adèrn B, Stenvinkel C, Sahlqvist L, Tegelberg ÅK (2014). Prevalence of temporomandibular dysfunction and pain in adult general practice patients. Acta Odontol Scand.

[4] Resende CM, Alves AC, Coelho LT, Alchieri JC, Roncalli AG, Barbosa GA (2013). Quality of life and general health in patients with temporomandibular disorders. Braz Oral Res.

[5] Dahlström L, Carlsson GE (2010). Temporomandibular disorders and oral health-related quality of life. A systematic review. Acta Odontol Scand.

[6] Akhter R, Morita M, Esaki M, Nakamura K, Kanehira T (2011). Development of temporomandibular disorder symptoms: A 3-year cohort study of university students. J Oral Rehabil.

[7] Velly AM, Look JO, Schiffman E, Lenton PA, Kang W, Messner RP (2010). The effect of fibromyalgia and widespread pain on the clinically significant temporomandibular muscle and joint pain disorders - a prospective 18-month cohort study. J Pain.

[8] Fernandes G, Van Selms MK, Gonçalves DA, Lobbezoo F, Camparis CM (2015). Factors associated with temporomandibular disorders pain in adolescents. J Oral Rehabil.

[9] Nilsson IM, List T, Drangsholt M (2013). Headache and Co-morbid Pains Associated with TMD Pain in Adolescents. J Dent Res.

[10] Ramalho D, Macedo L, Goffredo G, Goes C, Tesch R (2015). Correlation between the levels of non-specific physical symptoms and pressure pain thresholds measured by algometry in patients with temporomandibular disorders. J Oral Rehabil.

[11] Marklund S, Wänman A (2010). Risk factors associated with incidence and persistence of signs and symptoms of temporomandibular disorders. Acta Odontol Scand.

[12] Souza Barbosa T, Gaviao MB, Castelo PM, Leme MS (2016). Factors Associated with Oral Health-related Quality of Life in Children and Preadolescents: A Cross-sectional Study. Oral Health Prev Dent.

[13] Al-Khotani A, Naimi-Akbar A, Albadawi E, Ernberg M, Hedenberg-Magnusson B, Christidis N (2016). Prevalence of diagnosed temporomandibular disorders among Saudi Arabian children and adolescents. J Headache Pain.

[14] Sipilä K, Tolvanen M, Mitrirattanakul S, Sitthisomwong P, Järvelin MR, Taanila A (2015). Orofacial pain and symptoms of temporomandibular disorders in Finnish and Thai populations. Acta Odontol Scand.

[15] Futarmal S, Kothari M, Ayesh E, Baad-Hansen L, Svensson P (2011). New palpometer with implications for assessment of deep pain sensitivity. J Dent Res.

[16] Dworkin SF, Leresche L (1992). Research Diagnostic Criteria for Temporomandibular Disorders: review, criteria, examinations and specifications, critique. J Craniomandib Disord.

[17] Ciconelli R, Ferraz M, Santos W, Meinão I, Quaresma M (1999). Translation into Portuguese and validation of the generic questionnaire for quality of life assessment SF-36 (Brazil). Rev Bras Reumatol.

[18] Zucoloto ML, Maroco J, Campos JA (2014). Psychometric Properties of the oral health impact profile and new methodological approach. J Dent Res.

[19] Conti PC, Pinto-Fiamenghi LM, Cunha CO, Conti AC (2012). Orofacial pain and temporomandibular disorders: the impact on oral health and quality of life. Brazi Oral Res.

[20] Lim P, Smith S, Bhalang K, Slade G, Maixner W (2010). Development of temporomandibular disorders is associated with greater bodily pain experience. Clin J Pain.

[21] Huang Z, Lin X, Li X (2011). Characteristics of temporomandibular joint vibrations in anterior disk displacement with reduction in adults. Cranio.

[22] Racine M, Tousignant-Laflamme Y, Kloda L, Dion D, Dupuis G, Choinière M (2012). A systematic literature review of 10 years of research on sex/gender and experimental pain perception – part 1: are there really differences between women and men?. Pain.

[23] Machado LP, Nery Cde G, Leles CR, Nery MB, Okeson JP (2009). The prevalence of clinical diagnostic groups in patients with temporomandibular disorders. Cranio.

[24] Asakawa-Tanne Y, Su S, Kunimatsu R, Hirose N, Mitsuyoshi T, Okamoto Y (2015). Effects of enzymatic degradation after loading in temporomandibular joint. J Dent Res.

[25] Baker SR, Mat A, Robinson PG (2010). What psychosocial factors influence adolescents’ oral health?. J Dent Res.

[26] Fernandéz-de-las-Peñas C, Galán-Del-Río F, Alonso-Blanco C, Jiménez-García R, Arendt-Nielsen L, Svensson P (2010). Referred pain from muscle trigger points in the masticatory and neck-shoulder musculature in women with temporomandibular disorders. J Pain.

